# Electrowetting Performances of Novel Fluorinated Polymer Dielectric Layer Based on Poly(1*H*,1*H*,2*H*,2*H*-perfluoroctylmethacrylate) Nanoemulsion

**DOI:** 10.3390/polym9060217

**Published:** 2017-06-11

**Authors:** Jiaxin Hou, Wenwen Ding, Yancong Feng, Lingling Shui, Yao Wang, Hao Li, Nan Li, Guofu Zhou

**Affiliations:** 1 Institute of Electronic Paper Display, South China Academy of Advanced Optoelectronics, South China Normal University, Guangzhou 510006, China; houjiaxin_1993@163.com (J.H.); phoenix_wenwen@163.com (W.D.); fengyancong@m.scnu.edu.cn (Y.F.); shuill@m.scnu.edu.cn (L.S.); yao@buaa.edu.cn (Y.W.); 2 Shenzhen Guohua Optoelectronics Tech. Co. Ltd., Shenzhen 518110, China; nan.li@guohua-oet.com; 3 Academy of Shenzhen Guohua Optoelectronics, Shenzhen 518110, China

**Keywords:** Poly(1*H*,1*H*,2*H*,2*H*-perfluoroctylmethacrylate), nanoemulsion, spray coating, electrowetting, dielectric layer

## Abstract

In electrowetting devices, hydrophobic insulating layer, namely dielectric layer, is capable of reversibly switching surface wettability through applied electric field. It is critically important but limited by material defects in dielectricity, reversibility, film forming, adhesiveness, price and so on. To solve this key problem, we introduced a novel fluorinated polyacrylate—poly(1*H*,1*H*,2*H*,2*H*-perfluoroctylmethacrylate (PFMA) to construct micron/submicron-scale dielectric layer via facile spray coating of nanoemulsion for replacing the most common Teflon AF series. All the results illustrated that, continuous and dense PFMA film with surface relief less than 20 nm was one-step fabricated at 110 °C, and exhibited much higher static water contact angle of 124°, contact angle variation of 42°, dielectric constant of about 2.6, and breakdown voltage of 210 V than Teflon AF 1600. Particularly, soft and highly compatible polyacrylate mainchain assigned five times much better adhesiveness than common adhesive tape, to PFMA layer. As a promising option, PFMA dielectric layer may further facilitate tremendous development of electrowetting performances and applications.

## 1. Introduction

Since the first discovery in 1875, electrowetting has developed into an efficient tool to reversibly switch the surface wettability via applied electric field [1], and made tremendous progresses in electronic paper display [2,3,4], microfluidics [5,6], microlenses [7,8], fiber optics [9,10] and so on. Compared with early electrowetting-on-a-conductor, electrowetting-on-dielectric (EWOD) exhibits two outstanding advantages using the covering of hydrophobic insulator on electrode: (i) capability of lowering contact angle hysteresis to make surface microfluids move easily; (ii) capability of applying much higher electric field for great contact angle variation [11]. Hereinto this hydrophobic insulator is critical to the electrowetting effect of EWOD.

Till now, two groups of materials have been utilized as dielectric layer in EWOD. One is inorganic compounds and nanomaterials with high dielectric constant, i.e., SiO_2_ nanowire [12,13], silanized Si_3_N_4_ [14,15], ZnO nanorod [16,17], ZnO nanowire [18], ZnO inverse opal [19], ZnO/TiO_2_ nanofilm [20], surface fluorinated silicon nanosphere [21], and mesoporous silica [22] et al., These inorganic materials exhibit low driving voltage but their high polarity may induce weak hydrophobility and dielectric failure (including dielectric breakdown, ion permeation and charging effect). And difficult fabrication of thin film also limits their application. 

Another is hydrophobic polymers with low surface energy, i.e., Teflon AF [23,24], CytopTM [25,26], Parylene-C [27,28], polydimethylsiloxane [29,30,31], polyimide [24,32,33] and polyethylene terephthalate/polyethylene [34,35] et al., even photoresist Su-8 [36]. Their strong hydrophobility, good chemical stability and easy processing make them more and more common in EWOD, especially Teflon AF, Cytop and Parylene [37]. But they have obvious drawbacks, too. Take Teflon AF for example. Besides low dielectric constant [38], high price, bad adhesion, weak compatibility, organic solvent pollution (especially fluorinated solvents), tiny and numerous film defects (namely pin hole) [39] and so on are adverse to the lifetime, stability and cost of applied electrowetting products.

In this work, we introduced a fluorinated polymethacrylate—poly(1*H*,1*H*,2*H*,2*H*-perfluoroctylmethacrylate) (PFMA) to construct the dielectric layer of EWOD. Hereinto side fluorocarbon groups assign low surface energy and great hydrophobility, and polymethacrylate mainchains assign excellent flexibility, compatibility, plasticity and even relatively high dielectric constant to PFMA [40,41]. Particularly, some simple, green, efficient and low-cost preparation technologies, including emulsion polymerization and spray coating, are available to preparation on dense and ultrathin PFMA film. It is desperately favorable to improve the overall performances of dielectric polymer layer.

## 2. Materials and Methods

### 2.1. Materials

1*H*,1*H*,2*H*,2*H*-perfluoroctylmethacrylate (FMA; 97%) was purchased from J & K Scientific Ltd. (Beijing, China). Benzotrifluoride (BTF; 99.5%) was purchased from Acros. without further treatment. Sodium dodecyl sulfate (SDS; 99%) and potassium persulfate (K_2_S_2_O_8_; 99.5%) were provided from Adamas Reagent Ltd. (Shanghai, China). Dialysis tube (M.W. cut-off: 14 kDa; Shanghai Green Bird Technology Development Co., Ltd., Shanghai, China) was boiled in pure water for 10 min and stored in 1 mM ethylenediamine tetraacetic acid (EDTA) aqueous solution prior to use. All other reagents and organic solvents were of analytic grade and directly used.

### 2.2. Emulsion Polymerization of PFMA 

PFMA was synthesized according to the previously reported emulsion polymerization [42,43,44]. Typically, a predetermined amount of FMA (1.59 g, 2.99 mmol) was dissolved in 1 mL of BTF, and then uniformly emulsified with 7 mL of SDS aqueous solution (SDS: 66.3 mg) in a 50 mL centrifuge tube for 3 min using an ultrasonic cell crusher (VCX150PB, SONICS, Newtown, CT, USA). Under the protection of dry argon, 2 mL of K_2_S_2_O_8_ aqueous solution (K_2_S_2_O_8_: 15 mg) was added dropwise into the resulting emulsion and magnetically stirred at 94 °C for 24 h. The crude emulsion was dialyzed in deionized water for 3 days to remove residual small molecules. 

Afterwards, the resulting emulsion was mixed well with the saturated aqueous solution of sodium chloride (NaCl) in a 50 mL centrifuge tube for demulsification. The final PFMA nanoparticles were obtained by centrifugation (TGL-10B, Anke, Shanghai Anting Scientific Instruments Factory, Shanghai, China), vacuum drying (DZF-6020, Jinghong, Shanghai Jing Hong Laboratory Instrument Co., Ltd., Shanghai, China) and lyophilization (FDU-1200, EYELA, Tokyo, Japan) in turn.

### 2.3. Characterization of PFMA Nanoparticles

The morphology was photographed using scanning electron microscope (ULTRA55, Zeiss, Oberkochen, Germany) with the ions energy of 5 kV at 150 k magnifications. The dialyzed emulsion was diluted by 64 times with deionized water, and then dripped on copper platform and sputter coated with a thin layer of gold as sample.

The particle sizes and distribution were measured using nanoparticle sizing & zeta potential analyzer (NANO-ZS90, MALVERN, Malvern, UK). After dilution with deionized water, sample was added into polystyrene cell and performed at 20 °C.

The chemical structure was characterized using Fourier transform infrared spectrometer (FT-IR; VERTEX-70, Bruker, Ettlingen, Germany) in a spectral range of 4000–400 cm^−1^ with the resolution of 2 cm^−1^, and 600 MHz superconducting Fourier nuclear magnetic resonance (NMR) spectrometer (AVANCE IIITM, Bruker, Fällanden, Switzerland) in solid state.

The thermal properties were recorded using differential scanning calorimetry (Q20, TA, New Castle, PA, USA) under nitrogen gas (flow rate: 10 mL/min).

### 2.4. Preparation of PFMA Dielectric Layer

**Scheme 1:**
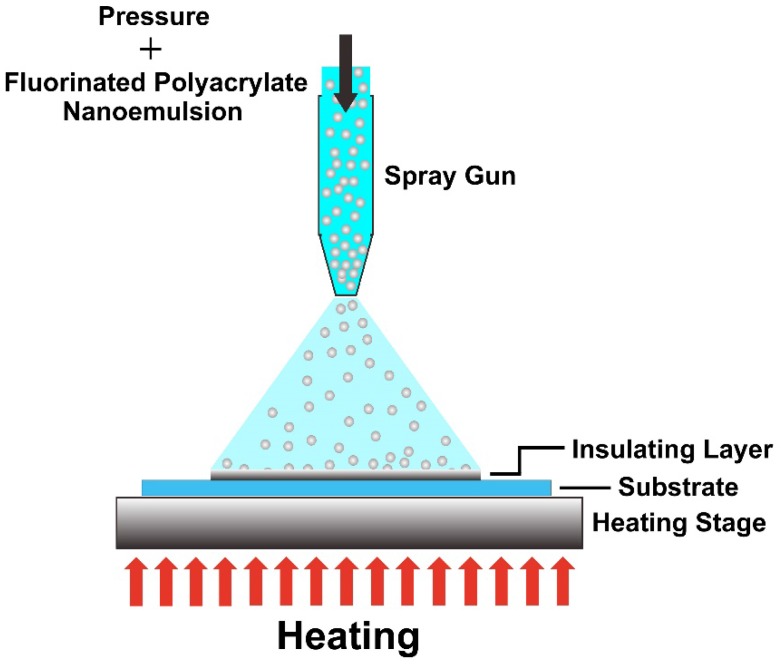
Schematic diagram of PFMA film preparation.

PFMA film was prepared according to the widely used technology—direct spray coating of aqueous nanoemulsion. Typically, the dialyzed emulsion was spray-coated on the indium tin oxide (ITO) glass via commercial spray gun (s-130, U-Star, Shenzhen, China; φ = 0.2 mm) with the working pressure of 15 psi and the air flow of 10.5 L/min (see Scheme 1). Here the heating temperature was set to the range of 80~110 °C, the distance between jet nozzle and ITO glass varied from 5 to 20 cm, and spraying time lasted from 10 to 60 s. Finally, the PFMA nanoparticles-coated ITO glass was heated to 210 °C for 15 min and then slowly cooled down to room temperature.

### 2.5. General Characterization of PFMA Dielectric Layer

The thickness was measured near by a bottom scratch on the surface of PFMA film using step profiler (Dektak XT, Bruker, Karlsruhe, Germany). The sectional topography was scanned using confocal microscopy (FV3000, Olympus, Tokyo, Japan). The transmittance in visible light range was determined using ultraviolet/visible/near infrared spectrophotometer spectroscope (UV/Vis/NIR; USB2000+, OCEAN OPTICS, Cincinnati, OH, USA). The surface wettability was evaluated by static and dynamic water contact angle. In detail, 10 μL of deionized water droplet was dropped onto the surface of the PFMA film from a hydrophobized needle of a micro-syringe. As the sample stage kept immobile or moving, the resulting contact angle was recorded by contact angle meter (JC2000C, POWEREACH, Shanghai, China) at ambient temperature, and measured by an image analysis software (DSA 10Mk2 drop shape analysis system, Krüss Hamburg, Hamburg, Germany).

### 2.6. Electrowetting Test of PFMA Dielectric Layer

**Scheme 2:**
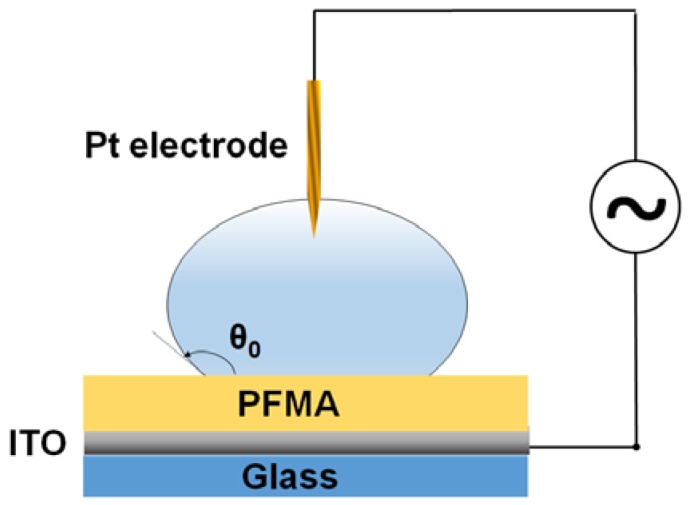
Schematic diagram of electrowetting test.

Just shown in Scheme 2, the positive pole of digital regulated direct current (DC) power supply (CE0400010T, Earthworm Electronics, Shanghai, China) was connected to the platinum (Pt) probe, and the negative pole was connected to the ITO side of glass substrate. Here the probe was inserted into a 10 μL droplet of 0.1 M NaCl aqueous solution on the surface of PFMA layer. As the applied voltage increased from 0 to 40 V with voltage step of 4 V, the real-time images of NaCl droplet were recorded using contact angle meter (JC2000C, POWEREACH, Shanghai, China), and measured using an image analysis software (DSA 10Mk2 drop shape analysis system, Krüss Hamburg, Hamburg, Germany).

### 2.7. Dielectric Determination of PFMA Dielectric Layer

The dielectric determinations were carried out using impedance analyzer (6500B, WAYNE KERR, Bognor Regis, UK). Same pole connection was performed just like the electrowetting test in Scheme 2. The resulting capacitance values and loss factors were displayed on the screen of impedance analyzer. Hence the relative dielectric constant (ε_r_) of PFMA film was figured out by the following equation:

(1) $$C=\varepsilon_{r}\varepsilon_{0}/t$$

where *C* is the capacitance per unit area, *t* is the thickness of the capacitor (i.e., dielectric layer between droplet and a solid electrode), *ε*_0_ is the permittivity of free space which is approximate to be 8.854187817 × 10^−12^ F/m. Particularly, the contact area between NaCl droplet and PFMA film was obtained according to the area calculation method of parallel-plate capacitor.

### 2.8. Breakdown Voltage (V_b_) Measurements of PFMA Dielectric Layer

Same pole connection of digital regulated DC power supply (CE0400010T, Earthworm Electronics, Shanghai, China) was performed just like the electrowetting test in Scheme 2. Along with increasing DC voltage, the real-time images of NaCl droplet were recorded by contact angle meter (JC2000C, POWEREACH, Shanghai, China) till one or few tiny bubbles were generating from electrolysis of NaCl droplet. At this time, the applied DC voltage was viewed as breakdown voltage of dielectric layer and the following.

### 2.9. Adhesive Force Measurements of PFMA Dielectric Layer

The adhesion determination was performed by pulling method using universal material testing system (LR10KPlus, LLOYD, Berwyn, PA, USA). In detail, polyimide (PI) was coated on the surface of ITO glass by spin-coating and then half of the PI layer was removed by half immersion in sodium hydroxide (NaOH) aqueous solution. Subsequently, PFMA layer was prepared on the surface of semi-covering ITO glass, and immersed completely in NaOH aqueous solution for the removal of residual PI as sample. Prior to determination, the glass substrate and the unadhesive half PFMA film were fixed on a couple of stretching clamps, respectively. The most common adhesive tape—scotch tape—was applied as control.

## 3. Results and Discussion

As an interfacial radical polymerization, emulsion polymerization can conveniently and continuously mass-produce water-based latex, namely aqueous dispersion of polymer nanoparticles or microparticles. However, it is not appropriate for condensation, ionic and other popular polymerizations. So, there are only a few options in dielectric polymers that are available for this green polymerization. Fortunately, PFMA is one such good choice. 

**Figure 1:**
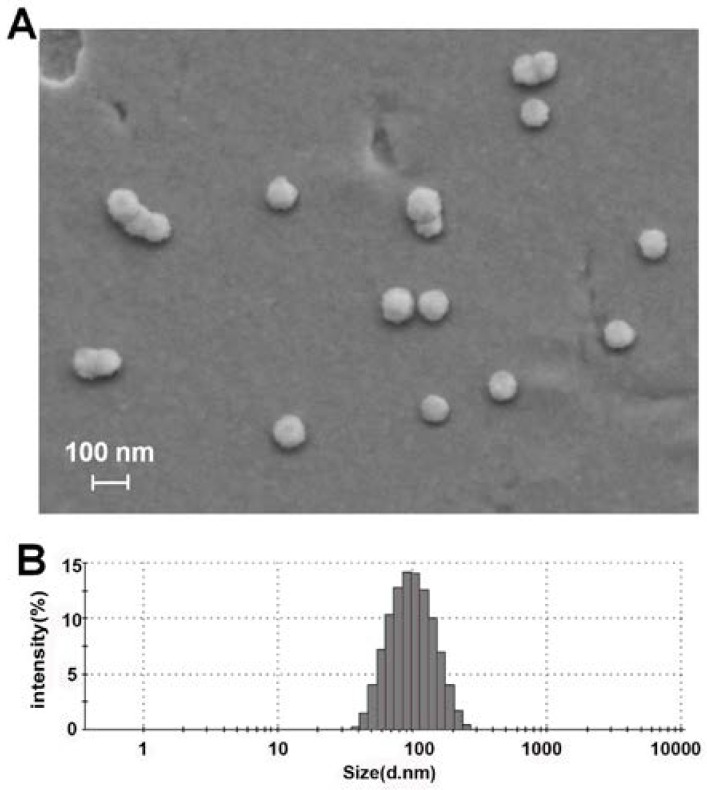
Morphology (**A**) and particle sizing distribution (**B**) of PFMA nanoparticles in the dialyzed emulsion.

At first, we prepared uniform PFMA nanoemulsion under the optimized polymerization parameters by ultrasonic emulsion polymerization. Figure 1A displays these spherical PFMA nanoparticles with similar diameter close to 100 nm. It corresponds well with the particle sizing distribution in Figure 1B. Here is only a single peak with narrow distribution and Z-average diameter of 92.173 nm.

**Figure 2:**
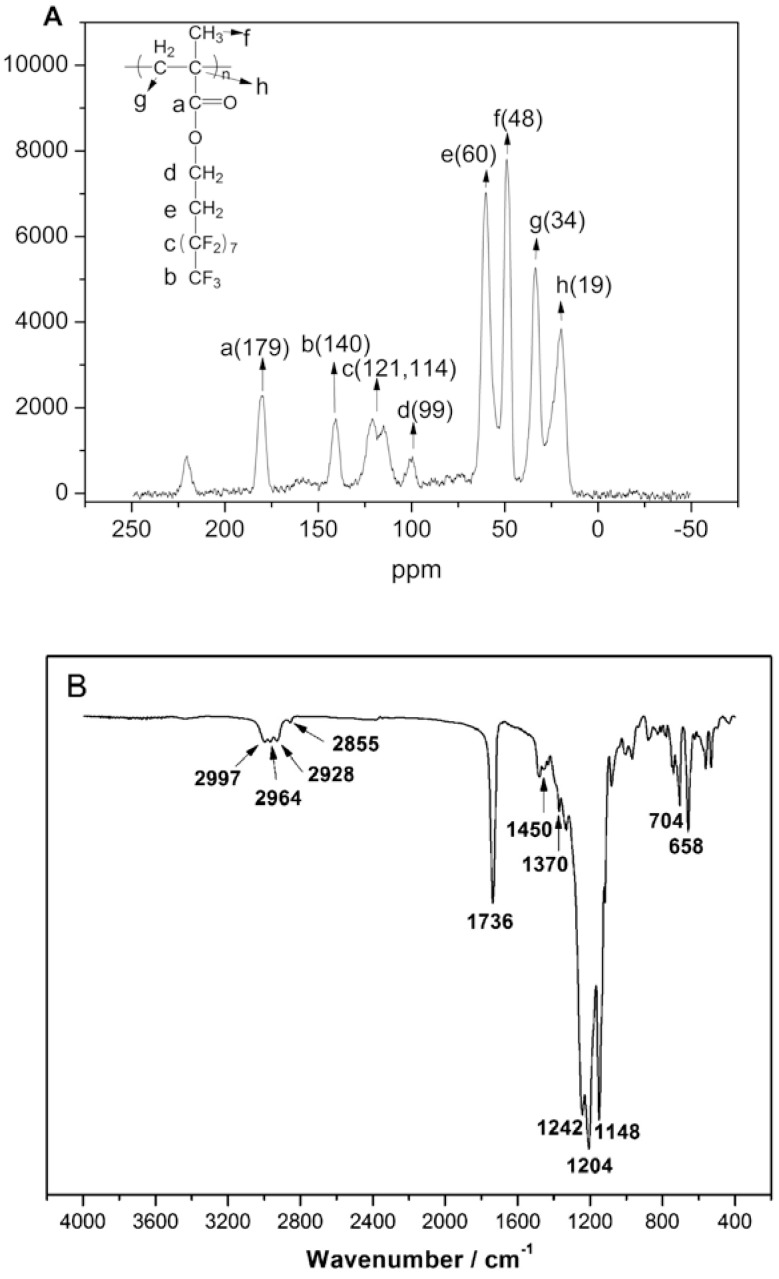
^13^C-NMR (**A**) and Fourier transform infrared (FT-IR) spectrum (**B**) of PFMA nanoparticles.

Figure 2A illustrates the whole chemical structure of PFMA by ^13^C-NMR spectrum. The only exception is the carbonyl carbon of residual FMA monomer presented at ~220 ppm due to p-π conjugation with the C=C of methacrylate. In detail, the peak at 179 ppm corresponds to the carboxyl group of the side chain, the peaks in the range from 16 to 48 ppm do to –CH_2_ and –CH_3_ groups of the main chain, and the splitting peak does to the –CF_2_ groups. Figure 2B provides more information of functional groups in PFMA listed below: 2997 cm^−1^ (CH_2_ linked with ester group), 2964 cm^−1^ (ω, CH_3_), 2928 cm^−1^ (CH_2_ linked with perfluoroalkyl group), 2855 cm^−1^ (CH_2_ in the polymethacrylate mainchain), 1736 cm^−1^ (ν, C=O in ester group), 1204 cm^−1^ (ν, C–O in ester group), 1450 and 1370 cm^−1^ (bending vibration of CH_3_), 1242 cm^−1^ (ν, CF_2_), 1148 cm^−1^ (ν, CF_3_), 704 cm^−1^ (bending vibration of CF_2_), and 658 cm^−1^ (bending vibration of CF_3_).

**Figure 3:**
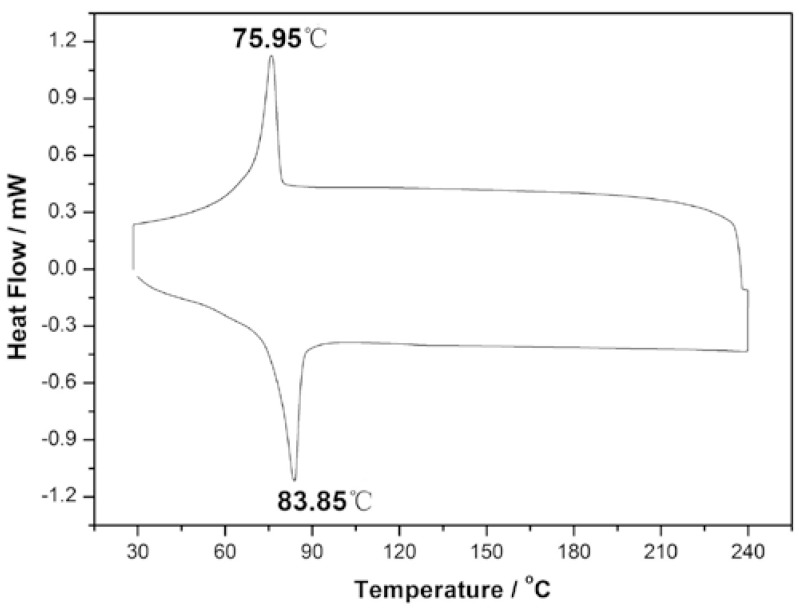
DSC curves of PFMA nanoparticles.

Figure 3 shows the thermal behaviors of PFMA nanoparticles. In detail, both the exothermic peak at 75.95 °C within the cooling process and the endothermic peak at 83.85 °C within the heating process testify their low melting point nearby 80 °C, which is consistent with the previous report [45]. It may originate from their own high surface energy and defects to decrease the required fusion heat greatly. This is a very favorable factor for film preparation, much superior to those common with high glass-transition temperature *T*_g_ and melting point *T*_m_ (i.e., Teflon series: *T*_g_ > 130 °C; *T*_m_ > 200 °C).

**Figure 4:**
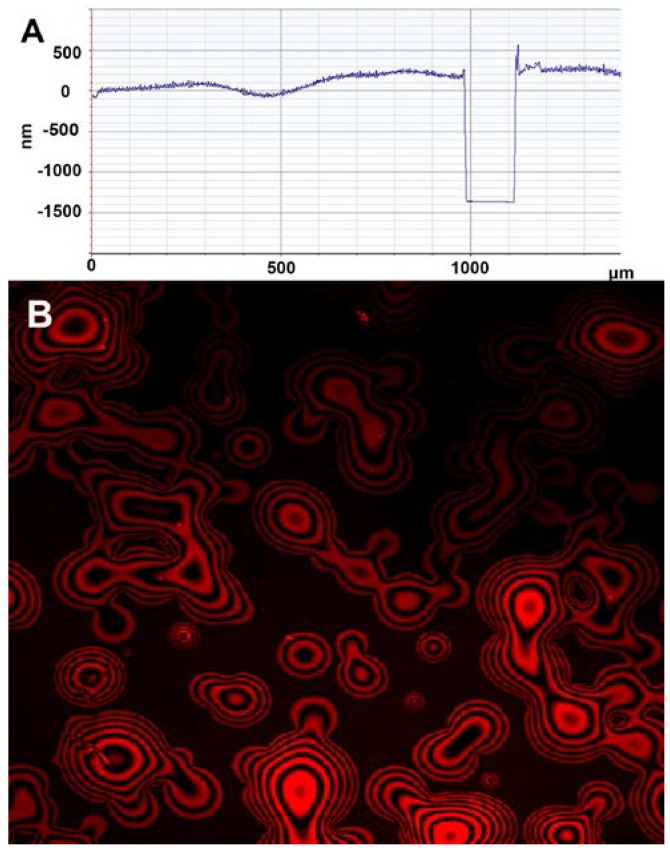
Step profile (**A**) and horizontal cross-section graph (**B**) of PFMA dielectric layer.

Therefore, we developed two heating stages during the process of PFMA film preparation: the first heating stage with the temperature range of 80~110 °C is to melt these PFMA nanoparticles for thorough film fusion; the second heating stage at 210 °C is to remove the residual water completely. However, it is still unable to ensure the forming of a perfectly horizontal and smooth surface. Just as shown in Figure 4A, the thickness of the PFMA film is about 1.5 μm but varied along with surface undulation. Fortunately, the local surface relief is usually less than 20 nm. So, the resulting PFMA dielectric layer is regarded as relatively smooth. Similarly, Figure 4B displays some multiple loopback-type traces left by the melting of PFMA nanoparticles, which also illustrates the undulating surface originated from the spray-coated particle distribution.

**Figure 5:**
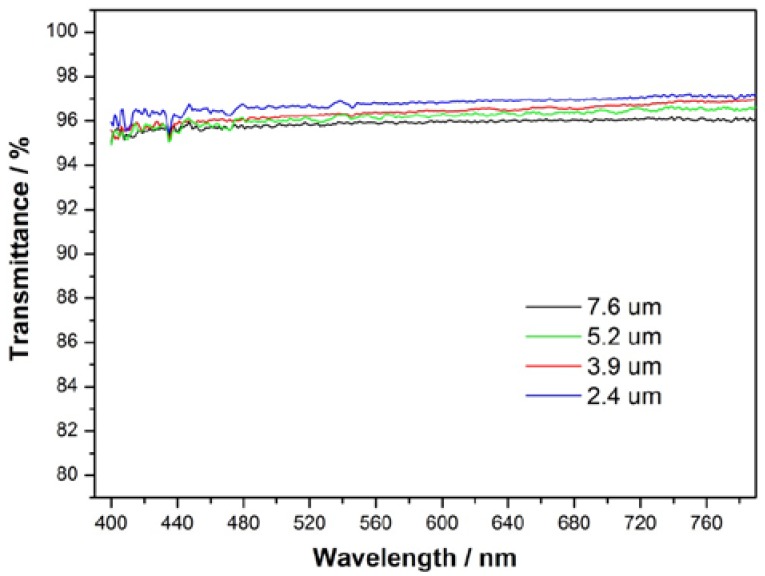
Light transmission of the PFMA dielectric layer with different thickness.

As we know, the light transmission of PFMA film is very important to optical applications, especially to electrowetting display. In Figure 5, PFMA exhibits excellent transmission that exceeds 95%, even though its thickness reached to 7.6 μm.

**Figure 6:**
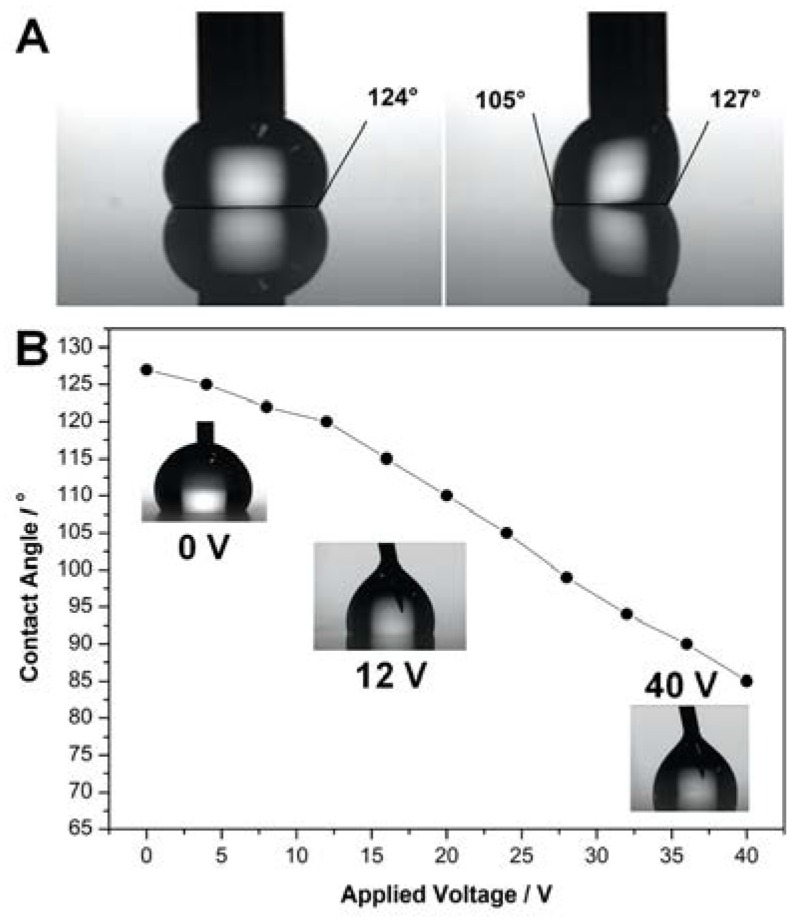
Surface wettability of PFMA dielectric layer: static and dynamic water contact angles (**A**), and the contact angle variation in applied electric field (**B**).

Furthermore, we evaluated the surface wettability and electrowetting effect of PFMA film by surface contact angle. As shown in Figure 6A, the static water contact angle reaches to 125° that is much higher than Teflon AF 1600’s (115°) and Cytop’s (90°). Apparently, this attributes to strong hydrophobility of PFMA. However, here there is an obvious difference between the advancing angle (127°) and the receding angle (105°). The contact angle hysteresis of 22° only results from the undulating surface. In Figure 6B, the contact angle obviously decreases from 127° to 85° till applied voltage rising to 40 V. Subsequently, the droplet keeps stationary no matter how high the electric field is applied. This reflects that, the contact angle has reached the saturation point that brings into a balance between surface tension and electrostatic force. Particularly, the over 40° contact angle variation is much better than other common dielectric polymers’, which may be relative to high dielectric constant of PFMA.

After all, the dielectric properties of dielectric layer are the most critical to electrowetting effect of EWOD. For example, low dielectric constant of Teflon AF 1600 (1.5~1.9) makes the dielectric layer easy to be broken down. Besides, plenty of unavoidable pin holes absolutely contribute to this dielectric failure (including dielectric breakdown, ion permeation and charging effect). Thus, we detailedly investigated three key characters in the dielectric properties of PFMA film: dielectric constant, loss factor and breakdown voltage.

**Figure 7:**
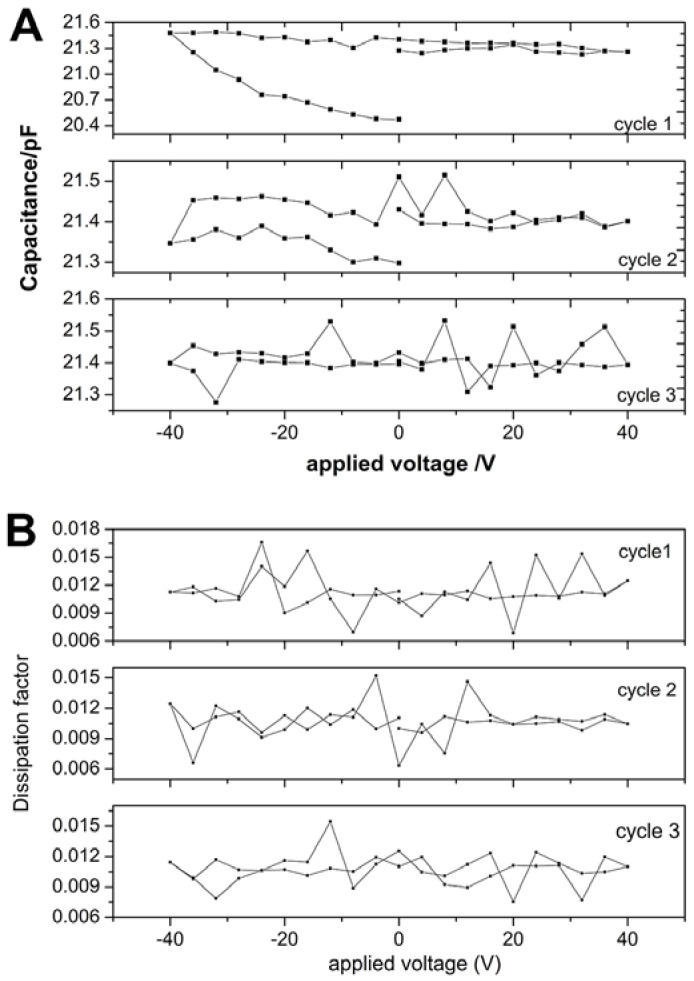
Capacitance (**A**) and loss factor (**B**) of the PFMA dielectric layer with the thickness of 1.4 μm at different applied voltages.

The dielectric constant represents a relative capacity for the storage of electrostatic energy in applied electric field. Figure 7A displays the capacitance curves of PFMA film in the range of applied voltage from −40 to 40 V. As the number of cycles goes up, the real-time capacitance value tends to keep stable. During this process of varying voltage, more and more charges will be gradually entrapped in the dielectric layer to fix the capacitance till saturated. Similarly, we also measured the capacitance values of other PFMA dielectric layer with different thicknesses (2.1, 2.4 and 3.9 μm). According to Equation (1), the dielectric constant value of PFMA is calculated to be 2.67, which is considerably higher than Teflon AF 1600’s (1.5~1.9).

Once the electric field is applied, part of the electric energy will gradually be lost or be converted into heat along with the polarization of dielectric molecules going on. Here the loss factor denotes the ratio value of the heat to the electric energy. Figure 7B displays the loss factor curves of PFMA film in the range of applied voltage from −40 to 40 V. Similar tendency appears during three same voltage cycles. Clearly, the loss factor remains at the extremely low level of approximately 0.011 in the three cycle tests. It reflects extremely low energy loss and high reversibility of wettability changing on the surface of PFMA film.

**Figure 8:**
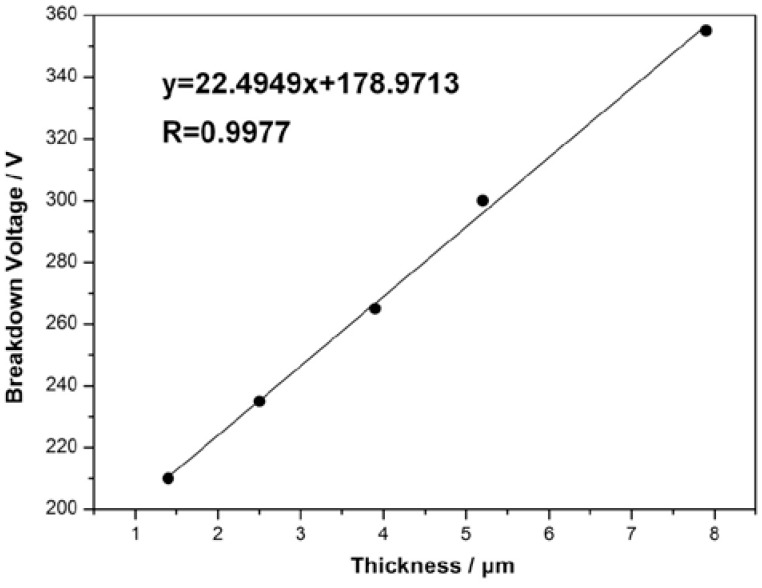
Breakdown voltages of the PFMA dielectric layer with different thicknesses.

In Figure 8, the breakdown voltages exhibit a highly linear relationship with the thickness of PFMA dielectric layers. According to the fitting equation, the initial breakdown voltage of PFMA is up to 179 V. If calculated based on the film thickness of 1 μm, the breakdown voltage will reach to 201.4662 V. In other words, the breakdown strength will reach to 201.4662 V/μm. Both of them are far higher than Teflon AF’s. This may arise from high compactness and dielectricity of PFMA film. No pin hole will greatly reduce the potential possibility of dielectric breakdown. 

**Figure 9:**
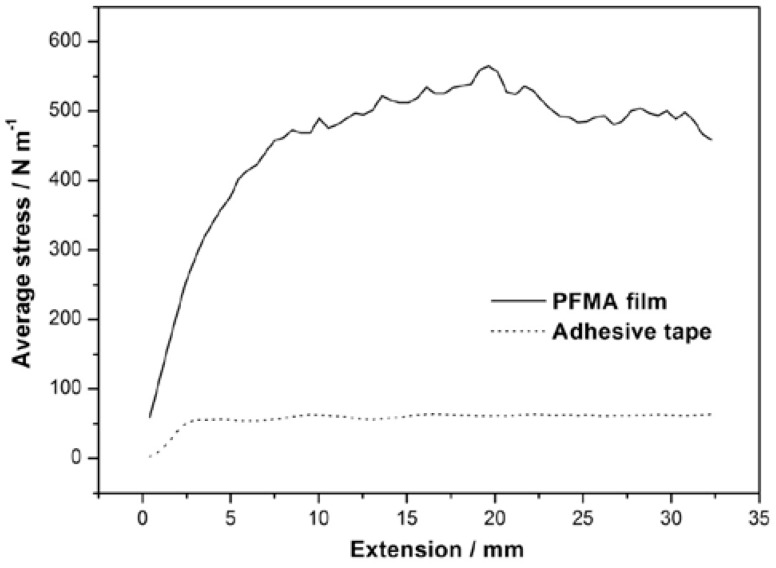
Stretch curves of the common adhesive tape (scotch tape) and the PFMA film with the thickness of 3.65 mm on the surface of indium tin oxide (ITO) glass substrate.

Moreover, bad adhesion of common dielectric polymers is also seen as a major defect in practical applications. If dielectric layer cannot tightly stick to the substrate, sliding, misplacing, bubbling, and even peeling are more likely to occur, leading to reduce the lifetime of EWOD devices. Particularly to the most common fluoropolymer, low surface energy and strong repulsion to other molecules does not favor the interaction with the substrate and other component. In order to avoid breaking during the stretching test, relatively thick PFMA film with the thickness of 3.65 mm was prepared as sample. Figure 9 shows much stronger adhesion of PFMA film to the ITO substrate. Significantly, the adhesive force reaches to 480 N/m, which is over five times higher than that of scotch tape. Moreover, as we know, Teflon AF 1600 layer is easy to be peeled off from the ITO substrate using scotch tape, indicating that the adhesion of scotch tape is much stronger than AF 1600’s. Undoubtedly, PFMA is also a fluoropolymer but possesses outstanding adhesivity.

## 4. Conclusions

In this work, we developed a novel fluorinated polyacrylate—PFMA dielectric layer for EWOD applications. Hereinto, both facile and green emulsion polymerization and spray coating were applied to construct continuous and dense PFMA film. All the results indicate that PFMA dielectric layer possesses relatively smooth surface, superexcellent light transmission, strong hydrophobility, and high dielectric constant of about 2.6. In applied electric field, PFMA layer behaves well with broad contact angle variation of 42°, extremely low loss factor of approximately 0.011 and high initial breakdown voltage of 179 V. Particularly, its strong adhesion is five times much higher than the common adhesive tape. All these dielectric and other key properties are better than Teflon AF 1600. As a dielectric material, PFMA may be considered as a good replacement for developing electrowetting performances and applications.
